# Sanatoria for Consumption

**Published:** 1905-06-24

**Authors:** 


### HOSPITAL ADMINISTRATION.

### CONSTRUCTION AND ECONOMICS.

### SANATORIA FOR CONSUMPTION.

LOCATION AND DESIGN.

Mr. Edwin T. Hall's Paper.

At the Parkes Museum on the 16th inst., Mr. Edwin T. Hall, architect, read a paper with the above title. Mr. Hall explained that these sanatoria should be distinctive in character and design, and distinct and separate in appearance from hospitals. He described them as centres for disseminating knowledge to patients and the public as to the fundamentals of sanitary living. They should be schools of hygiene, where the practical value of sunlight and fresh air, regularity of living, exercise, cleanliness and orderliness are taught and demonstrated. Dealing with their location and design he defined the best site as the southern slope of a gentle hill near its summit, sheltered by trees on the north, east, and west. The sub-soil should be sand or gravel, because it is dry and warm, and in any case a naturally well-drained site is imperative. Mr. Hall next dealt with water supply, sewage disposal, the value of trees for shelter, a wide and extensive prospect, and a southern aspect. He regarded the welfare of the patients as the first consideration, and next the convenience of the administration. Mr. Hall then gave a short account of the principal foreign sanatoria. The hotel or barrack unit is the chief type in Germany, France, and Switzerland, where the sanatoria are from three to five storeys high. The keynote in the German sanatoria is concentration with centralisation for purposes of economy.

Some German Sanatoria.

Mr. Hall exhibited the plans of most of the following sanatoria:—

*The Falkenstein Sanatorium*

is in effect a large hotel, which began from a small private mansion, but has been added to at different times. The kitchens, larders, and offices are on the basement of the central building—U-shaped on plan, the arms being nearly at right angles to the centre. This block faces E.S.E. It is four storeys in height including the basement, and there are three main staircases. To the N.E. is a very large dining hall opening on the S.S.E. to a wide verandah, which is extended to form a covered promenade 200 feet long. The other corresponding wing has an enclosed gallery facing west. The medical staff houses and consulting rooms are at the extreme ends. There are no regular nurses, but the whole plan is under medical direction, and accommodation is provided for 120 patients of either sex, in 70 single and 25 double-bedded rooms. Most of them have south aspects, but many have not. There are only two slipper-baths for patients and a douche room. The Liegehallen are attached, forming wide verandahs on the three sides of the terrace at the basement floor level, and in the grounds there are several open pavilions and summer houses.

*Ruppertshain*

is a compact building, crescent in form, consisting of a basement with four storeys above in which the administrative departments and patients' room are all contained. It accommodates 112 patients in wards containing one, four, and six beds each. These rooms are in single file and face south, with a corridor on the north side. There are two main staircases. There are nine slipper-baths and one douche room for men, another for women. There are a matron and five nurses The Liegehallen are two-storeyed, one on each side, attached as wings.

*The Nürnberg Heilstätte at Engelthal*

is one building with basement and four other floors. Here again the administrative departments are in the same block as the patients. Fifty patients are accommodated in 17 bedrooms; two only have one bed, others three and five beds each. All face south. There are three nurses. There is but one staircase in the building, which would hardly pass our fire-escape authorities. There are four slipper-baths and three douches, all in the main building, as are also the six w.c's. The Liegehallen are detached. The last two institutions described are, it will be seen, of the one block type, but the next is quite different.

*The Städtisches Sanatorium at Harlaching near Munich*

is, to be accurate, a hospital where the large majority of patients are consumptive. The main block is E-shaped, three storeys in height, with no basement. It receives 212 patients, 106 of each sex in 28 wards, 12 with single beds, six with 12 beds, and six with 20 beds. There are besides a few isolation-wards. There are four staircases and one passenger-lift. There are 12 slipper-baths and three douches divided into six groups, each having two slipper- and one douche-bath in a single room divided by partitions. There are 18 w.c's, each group of three with a slop-sink being in one room with one window, and all in the body of the building. The 20-bed wards are axially east and west, the 12-bed wards north and south, all with windows on both sides. The single rooms face east or west, and the isolation-rooms north. The Liegehallen are three storeys high, forming 12 feet wide verandahs of solid masonry on the south side of the 20-bed wards, which are thus practically cut off from all sun. There are other Liegehallen in the grounds. There are two very handsome chapels in this hospital. The nursing staff consists of 23 and a matron. The administration block is separate, and is a hollow square on plan, with the engine and boiler-houses in the centre of the quadrangle. It contains the staff residences, the kitchens, staff dining-rooms, laundry, cow stables, &c. The disinfector is, strangely, placed in the basement of the staff block. It will be seen that this very modern and complete hospital disregards what we consider of great importance—viz., aspect, abundant sunlight in all rooms, and detachment of sanitary apparatus.

*The Volksheilstätte at Krailing, in Bavaria,*

is more like our accepted type than any other, but it has a basement and three storeys. Its plan consists of a centre with two wings at very flat angles. It takes 120 patients. There are 13 single-bed wards, the others containing two, three, four, five, and six beds, all facing S.S.E. or S.S.W. There are two staircases. Four slipper-baths are provided in one room, and there is one douche room. Here again the 18 water-closets are in groups of three in one room with one window, and all are in the heart of the building. There are also in the body of the building rooms fitted with 30 lavatory basins. The nursing staff consists of 15. The staff all live in this same building. The Liegehallen are recessed verandahs under the patients' bedrooms of the side wings.

Mr. Hall next dealt with the King's Sanatorium, that at Norwood, and the Heatherside Sanatorium, Frimley. We have already published plans with a full description of the last-named, and hope shortly to give the same information about the first two.

Mr. Hall's Views.

Mr. Hall favoured large buildings, with wards containing one to three patients, rather than those with separate chalets or cottages, of which latter Nordrach in the Black Forest was the chief type. The question in England to-day was how to erect cheap sanatoria for the million, a problem yet to be solved. Mr. Hall maintained that it was impossible to get buildings of a permanent character, with all the modern minutiæ of sanitary detail insisted upon by experts, without great cost. He is evidently in favour of incurring this cost and did not attempt any practical solution of the larger question. He appears to think that, should this type of buildings in a few years turn out to be unnecessary, owing to the advances in treatment, expensive sanatoria costing £400 to £600 per bed could be utilised for some other purpose.

### The Discussion.

The discussion which followed was disappointing. It contained very little in the way of practical suggestion and ended in a flood of sentiment for the enforcement of the impossible view that great homes for the dying should be erected throughout the country by local authorities in which patients suffering from tuberculosis should be maintained until death put an end to their sufferings.

Mr. Scovell (Metropolitan Asylums Board) said he came there to learn. He pointed out that the choice of site in the case of sanatoria was much wider than in the case of fever hospitals. He was sorry that Mr. Hall offered authorities no encouragement in the direction of a reduction in the cost of buildings. He quite agreed that the first cost in a properly planned building proved in practice the least cost for hospitals of a permanent character. He feared there was small prospect of reduction in the cost of buildings, though it was essential to secure it to the utmost practical extent.

Mr. Percy Adams, the architect of the King's Sanatorium, agreed that these buildings should resemble in character that of a country house with a cheerful aspect and situated in attractive grounds. The southern aspect was the best, but at Davos a northern aspect had been chosen. He referred to a new washable paper, which was so constructed that it could be washed frequently for 12 years without forfeiting its sanitary efficiency. He mentioned that the wards in the King's Sanatorium were to be heated from below the floor. Novel as Mr. Hall's plan was, he understood that a building of the same general plan had been erected in Chili.

Dr. Theodore Williams congratulated Mr. Hall on striking out a line for himself. Chili, he said, was a long way off, and Mr. Hall might claim originality for his design. The site should be elevated and sunny; it should face the right aspect, and the buildings should contain an abundance of electric light. In order to justify the erection of sanatoria they must provide what the patients cannot obtain elsewhere, and secure to them an abundance of sunshine and an abundance of fresh air. He enforced the view that the treatment of these cases in tents and huts had produced good results. The cottage form was good, but in practice amounted to the same thing as that of a larger building. On the cottage plan they must have an administration building, and on the pavilion plan an administration building with corridors of cottages. He supported the erection of large buildings with facilities to shut windows and doors and prevent draughts. The power block should be well away from the main buildings. He was strongly of opinion that the initial cost of building was at present too great and should be reduced. It ought to be and it could be reduced. In Germany sanatoria cost about £200 per bed against from £400 to £600 per bed in England. In Denmark he found that large sanatoria for the people had been erected at a cheap rate—the two largest cost about £187 per bed. In France, where these sanatoria with 120 beds contained everything that was necessary, the cost was £134 per bed. In Switzerland, too, the buildings were cheap, and also in America where asbestos buildings were favoured. Germany, of course, had some very expensive sanatoria. In England we required more beds for tubercular cases, especially for the poor, and they could only be provided in relatively cheap buildings by local authorities.

Mr. Aldwinkle said the difficulty of the provision of accommodation for the poorer classes was primarily one of cost. It was quite impossible for every public authority to provide two or three hundred beds for tubercular cases at a cost of from £400 to £600 per bed. It was essential to find some new method of construction, for without it little reduction in cost could be hoped for. A brick building of the cheapest kind meant a cost of £300 per bed. Why not try wood and half-brick construction, covered with felt or asbestos? Buildings of this character, being light, needed no costly foundations, and if they were deliberately undertaken and plenty of time allowed for their erection, they could be provided with proper sanitary arrangements, and be made in every way suitable at relatively small cost. He mentioned that two light hospital buildings were already in existence and had proved successful. Either the cost of erecting sanatoria must be cheapened, or the movement in favour of their erection must die out.

Dr. Heron went to the root of the matter by condemning in suitable language the hard measure meted out to members of the poorer classes when suffering from tuberculosis. He stated, as the result of his experience as a hospital physician, that the needs of the moment were adequate accommodation for the prompt isolation and treatment of the breadwinner when attacked by this disease. In existing circumstances he gets an out-patient letter, is examined by the physician and prescribed for, and given some papers which inform him that he is suffering from a contagious disease, and that he should not associate with members of his family. His earnings have never been large, and his condition of health renders it impossible for him to continue to work or to provide himself with the open-air treatment under favourable conditions without which he must speedily grow worse. He goes home from the hospital, studies his papers, realises the impossibility of securing the necessary treatment, knows that his small savings must speedily be exhausted, and that his family will soon sink to a condition of destitution. How is the illness of such a man in depressing circumstances like these, and in the absence of adequate accommodation provided by the public authority, to avoid sinking to a state of misery and depression, which must tend to hasten his death? Dr. Heron stated that in round figures there were 50,000 deaths from tuberculosis in this country each year. He estimates that there must be some 250,000 people suffering from tuberculosis, and that of this number two-fifths, or 100,000 are breadwinners of the poorer classes. What was needed was some cheap form of construction which would encourage municipalities and county councils to provide buildings for the accommodation of the humbler citizens suffering from tuberculosis. It was monstrous to expect that the ratepayers could provide the money to erect large sanatoria at a cost of anything like £500 to £600 per bed. The cost ought to be reduced to something like a third of this sum, and he expressed some confidence that this could be done if proper steps were taken to solve the problem. Dr. Heron mentioned as types of cheap buildings those at Banstead, the Grove Farm Small-Pox Hospital, Colney Hatch, and Hanwell. At the two latter the cost of buildings had been about £80 per bed, and at Banstead £50 per bed. These buildings would last 20 years and not cost much for repairs. He had questioned the residents, both patients and staff, and found that all preferred temporary to permanent buildings as places of residence. He considered the present common type of treatment of tubercular cases at hospitals as a scandal, and he supported Dr. Williams's views as to the essentials.

The discussion was continued by Dr. Squire and others, and lasted till a late hour.

### The Heatherside Sanatorium, Frimley.

A visit was paid to this institution on Saturday the 17th inst. It is not necessary for us to repeat the details and plans of this institution, which were published in The Hospital of June 25, 1904, pp. 230 and 231. The buildings present an attractive appearance. Mr. Edwin Hall has evidently taken infinite pains to introduce the minutiæ of sanitary detail insisted upon by experts, and the result is a very costly building, charming in many aspects, and one which should prove suitable for the treatment of tuberculosis, providing the pavilion is the best form of erection for this class of case. Our views on this point are well known, and we cannot do better than repeat what we said a year ago when describing the plans of the Heatherside Sanatorium.

"As already said, we look upon the plan as a good one provided it be thought desirable to accommodate so many patients under one roof, even if in sections so well separated as these are. Probably the architect had not much choice on this point, but we believe that in these sanatoria for consumption the development will be towards a much smaller unit than 11 patients—will, in fact, be towards single-bedded huts properly constructed and entirely separated from their fellows."

In other respects the justice of the criticisms which we made on the plans are confirmed by a personal inspection, and whilst we congratulate Mr. Hall upon a successful piece of work, we could wish that he had set himself to act as a pioneer in sanatorium construction by planning a building and erecting it at a cost of something like one-third of the actual sum which has been expended at Frimley.

